# Hair cortisol concentrations in clipped and combed hair and associations with characteristics, health status and stress in domestic cats

**DOI:** 10.1038/s41598-024-73226-w

**Published:** 2024-09-19

**Authors:** Ninni Rothlin-Zachrisson, Helena Röcklinsberg, Emma Jettel, Felicia Johansson Bergqvist, Sarah Stadig, Malin Öhlund, Chiara Mariti, Bodil Ström Holst

**Affiliations:** 1 Department of Clinical Sciences, Swedish University of Agricultural Sciences, P. O. 7054, 750 07 Uppsala, Sweden; 2 Department of Animal Environment and Health, Swedish University of Agricultural Sciences, P. O. 7068, 750 07 Uppsala, Sweden; 3 Falköpings Smådjursklinik, Agnestadsgatan 3, 521 40 Falköping, Sweden; 4 Swedish Medical Products Agency, P. O. 26, 751 03 Uppsala, Sweden; 5 Department of Veterinary Sciences, University of Pisa, Viale Delle Piagge 2 I-56124, Pisa, Italy

**Keywords:** Cats, Noninvasive measures, Cortisol, Chronic disease, Estrus, Pregnancy, Physiology, Endocrinology

## Abstract

Hair cortisol concentrations (HCC) are measured to assess long-term HPA-axis activity and may represent a valuable non-invasive tool to evaluate chronic stress in cats. This study investigated combing as a novel, low-stress method for HCC assessment, as well as possible associations between HCC and cat characteristics in 167 owned cats. Hair was sampled at veterinary clinics through clipping and/or combing the cat, or at home by the owner combing the cat. A questionnaire was sent to cat owners, including inquiries about the cat’s sex, health status, and exposure to stress. HCC was quantified using a commercial cortisol assay kit. Despite variations within and between sampling methods, Spearman’s correlation and Bland–Altman plots revealed a moderate correlation between clipped and combed samples (rs = 0.61, LOA -5.51 ± 22.54). In multiple linear regression, variations in HCC were observed based on sex, health status and cat group size. No associations were found between HCC and stress as assessed by owners. Despite study limitations and remaining uncertainty regarding factors influencing HCC, combing presents a convenient approach for evaluating long-term HPA-axis activity in clinical settings. The association between health and HCC suggests alterations in cortisol levels that are related to disease processes and stress-inducing events associated with the disease.

## Introduction

Chronic stress is a critical concern for animal welfare^1–3^, drawing increased attention in association with different physiological and behavioral alterations in cats^4–10^. Despite its significance to both cat and owner, signs of stress are frequently overlooked or underestimated by owners^11^. Stress is best estimated by combining behavioral and physiological data^12,13^, and as the hypothalamic–pituitary–adrenal (HPA) axis is central in stressor responses, measurement of HPA-axis activity is a common approach.

Over the past two decades, the use of hair cortisol concentration (HCC) to evaluate long-term HPA-axis activity has gained prominence^14–18^. HCC, reflecting cortisol incorporation into actively growing hair, is regarded as a reliable marker for long-term cortisol secretion in various species^19^. The benefits of using HCC include an easy and non-invasive sampling, convenient storage of hair^20^, and an excellent stability of cortisol in hair over time in investigated species^18,20–22^. Striving for a low-stress and convenient sampling method is beneficial for both cats and their owners. Traditionally, hair for analysis of cortisol concentration is sampled through shaving, often using the shave-reshave method, where hair is shaved at the beginning of the time frame of interest, and then shaved again after a period of regrowth^23^. Shed hair has been collected from sleeping nests^24^ and from fencing^25^.

However, our understanding of HCC has limitations, and recognizing them is essential, particularly when intending to evaluate animal welfare and stress. The mechanisms of cortisol incorporation into the hair are not fully understood^23^, and both hair and species-specific factors have to be considered when evaluating HCC^18,22–24,26–32^. In domestic cats this includes the varying distribution of primary and secondary hairs and seasonal hair follicular activity^33,34^. Higher HCC have been associated with litterbox issues^35,36^, aggressive behavior^36,37^, and neutering status in cats^37^, and both physiological and behavioral alterations have been suggested to affect cortisol concentrations in reproductively active females^37,38^. Lower HCC was measured in cats with well-kept hair coats compared to cats with poor hair coat conditions ^35^. An unkempt hair coat is reported in association with stress^39^ and with chronic diseases like hyperthyroidism and diabetes mellitus in cats^40–42^. However, when considering other factors, owner-reported chronic diseases in cats did not reveal significant associations with cortisol concentrations in either hair or nails^35^. Higher HCC were measured in shelter cats with dermatophyte infections^43^, but the only other factors available for data analysis were age and sex. This study aims to investigate a new low-stress hair sampling method in cats, building on previously used hair clipping techniques^35–37,43,44^, by combing the thoracic and abdominal regions. The objective was to develop a method that minimizes the need for sampling equipment, simplifies recruitment by lowering barriers for cat owners, and ensures low-stress handling of the cats. Another aim was to evaluate HCC in relation to owner-reported cat characteristics, health status, stressors, and signs of stress. We hypothesize that HCC from different sampling locations, sampled using different methods, will be positively correlated, and that HCC will be influenced by chronic disease and stress.

## Materials and methods

### Ethical approval

According to Swedish (SJVFS 2019:9, case no. L 150) and EU (EU Directive 2010/63) legislation, hair sampling by combing or clipping from privately owned cats does not require ethical permission. As required by L150, the owners received both written and oral information about the study and signed informed consent prior to inclusion. They were informed that they could withdraw their consent at any time. The experimental protocol was approved by The Board for Animals in Research and Teaching at The Swedish University of Agricultural Sciences. Data were handled in accordance with the General Data Protection Regulation. Hair samples were the only samples collected from the cats in this study, and all sampling took place in conjunction with veterinary appointments or by the owners at home. This study is reported in accordance with the ARRIVE guidelines^45^.

#### Study sample and questionnaire

Cats were recruited, and hair was sampled during veterinary visits to four participating veterinary clinics. Cats were also recruited through personal contacts, and in these cases, hair was sampled by the owners at home. Recruitment was limited to cats aged ≥ 6 months living in Sweden, whose owners consented to their participation. Participation was entirely voluntary, and no remuneration was provided. Information about the cats was obtained from a questionnaire. The exclusion criteria encompassed an incomplete questionnaire, contradictory information on health status of the cat, and the presence of acute injury, acute disease, or treatment with glucocorticoids 2–16 weeks before hair sampling.

Questions for the questionnaire were formulated taking into account previous studies and knowledge on stress in cats and the role of the owner in assessment^3,7,11,46–48^. The questions were assessed for comprehensibility and accurate interpretation by four cat owners, who were not part of the study. Based on the feedback from this pilot testing, the questionnaire was revised before digital distribution to participating cat owners via an online provider (Netigate), by e-mail, or, in case of missing e-mail, by mail.

Most questions were related to the time frame 2–16 weeks before hair sampling, roughly representative of HPA activity and cortisol incorporation in sampled hair strands. In addition, some questions concerned basic information about sex (male or female), the cat’s age at hair sampling, hair color and pattern. Further, two inquiries were about the owners’ perception about the cat’s quality of life and welfare. All owners had to answer the question “Is your cat healthy?” with answer options “Yes, my cat is healthy”, “Yes, my cat is healthy but has suffered from acute disease or injury”, “No, my cat has a chronic disease” and “No, my cat has a chronic disease and has suffered from acute disease or injury”. This formed the basis for grouping the cats according to their owner-reported health status. To assess the presence of stress, information was gathered on owner-reported exposure to potential stressors (e.g., new animal in household, house renovation, or cat relocation) and owner-observed signs of stress (e.g., house soiling, increased conflicts with other animals, changes in appetite or in activity levels). For cats with chronic disease, information on the presence of clinical signs related to the disease was gathered as a measure of disease impact on affected cats. For a summary of the questions and answer options, refer to Table 1. The answers from twenty-five questions were analyzed in this study.

**Table 1:** Summary of merged questions and answer options, as they were grouped for data analysis, as completed by owners of cats encompassed in the study (n = 167).

Questions	Answer options and grouping of answers for data analysis (all questions included an “Other/Decline to answer” alternative and a free text section)
**Cat characteristics**	
Year of birth	e.g., 2014
Sex and neutering status	Spayed female, Neutered male, Intact female, Intact male, Female with experience of estrus/pregnancy
Color pattern*	Tabby/striped, Tortoise/patchy, Full colored, Masked, Other
Main color*	Black, White, Grey, Brown, Red, Other
Hair length	Short, Semi-long, Long
**Living conditions**	
Living location	Centrally in a city, Outside city center (e.g., residential area), In a smaller town, In the countryside
Cat age when arrived at owners home	Introduced as a kitten, 6–12 months of age, Older than 12 months
Presence of children < 18 years of age in household	No, Yes
Outdoor or indoor confinement	Indoor (strictly indoor and indoor with access to patio/balcony/leash walks), Outdoor (both out- and indoor and strictly outdoor)
Number of cats in household	Single cat household, Multi-cat household
Presence of other animals in household (e.g., dog)	No, Yes
Harmony in animal group	Occasional conflicts or occurrence of cat withdrawal, Many conflicts or occurrences of cat withdrawal, No the animal group is harmonious, No other animals in household
**Health status**	
Presence of chronic disease	Yes (Hyperthyroidism, Diabetes mellitus, Chronic kidney disease, Other), No
Presence of clinical signs related to chronic disease?*	No, Yes (Polyuria/polydipsia, Changes in appetite, Weight loss, Weight gain, Other)
**Welfare and stress**	
Exposure to potential stressors*	No, Experience of potential stressors (Moved to a new home, Big changes in family/household (e.g., new family member), House renovation, Travels/relocation of the cat, New animal in household, Move or death of former animal friend, Diet change, Other)
Display of signs of stress*	No, Displaying signs of stress (House soiling, Altered grooming activity, Behavioral changes, Increased occurrence of conflicts/aggressive behavior, Scratching furniture, Signs of withdrawal, Changes in appetite, Reluctant to play, Spending more time outdoor/indoor, Seeking more contact, More vigilant, Strong reaction when scared, Other)
**Other**	
Treatment with glucocorticoids	No, Yes (Oral tablets, Salve, Ear drops, Injection, Other)
Treatment with other medications or supplements	No, Yes (Free text answer on what medication)
*Multiple answers available	

#### Hair sampling

All hair sampling occurred between June 2020 and November 2022. Hair was sampled on one occasion from every cat. At least one hair sample was obtained from each cat: by clipping the dorsal area of the front leg or the caudoventral area of the abdomen, or by combing the dorsal back and lateral sides of the cat. Hair sampling was conducted either at veterinary visits (both clipping and combing) or by owners at home (combing only). For owners visiting veterinary clinics, paired samples were taken from more than one of the three different locations and methods. Clipping areas were selected for ease of retrieval, representing common venipuncture sites (dorsal area of the front leg) or areas prepared for abdominal ultrasound or surgery (the abdomen). This also ensured that no cat had to be clipped when not medically indicated, creating an opportunistic sampling process.

Detailed written instructions were provided to both veterinary staff and owners before sampling. Front leg and abdominal samples were obtained using well-cleaned mechanical clippers designed for small animals. The hair was cut at the level of the skin surface. For the front leg, a square of hair approximately 1.5 × 1.5 cm was cut from the dorsal area of the radius. Abdominal samples involved clipping hair from the caudoventral abdominal area in the anterior direction towards the costal arch, covering an area of approximately 5 × 10 cm, depending on the cat’s size. Instructions for combing included using a fine-toothed, cleaned comb on the dorsum and lateral area of the cat’s thorax and abdomen, and combing until an amount of hair equivalent to approximately 1 cm in diameter, when compressed, was achieved.

The hair samples were assigned a code for blinding, were placed in aluminum foil, and were stored at room temperature in a dark, dry place for a maximum time of 7 months before cortisol quantification.

#### Cortisol extraction

All laboratory work was performed by the research team, at the laboratory at the department of Clinical Sciences, Swedish University of Agricultural Sciences, Uppsala, Sweden. Based on previously measured hair growth rates in domestic cats^34,49^, the sampled hair was cut to a length of 40 mm, estimated to roughly represent the hair growth during 16 weeks. Extraction of cortisol from hair was performed and partly modified after Meyer et al.^15^, as follows: roughly 50 mg of hair was clipped into smaller parts with scissors and washed twice in 1.5 ml of isopropanol using a vortex mixer. When dried, excess hair was removed for a final hair weight of 50 mg. For samples containing hair < 50 mg, the lower weight was noted. In preparation for grinding the hair, three steel balls (Ø3.2 mm) were added to each sample and samples were deposited in liquid nitrogen for 4 min, increasing hair brittleness. Hair grinding was performed using a BeadBeater (5000 RPM, 6 × 30 s with a 30 s pause between sets). Freezing and grinding were repeated once. For cortisol extraction, 1.2 ml of methanol was added to the ground samples in a 22 h incubation phase on a laboratory rocker. After centrifugation, 0.6 ml of supernatant were extracted from each sample and then dried and reconstituted in a phosphate buffer (0.01 M PBS, pH 7.4). Reconstituted samples were stored frozen (-80°C) until cortisol quantification.

#### Assay performance and determination of cortisol concentration

For quantification of cortisol in hair, a commercial competitive high sensitivity ELISA kit (Salimetrics® Salivary Cortisol Enzyme Immunoassay Kit, LCC 2021) developed for quantitative measurement of human salivary cortisol and previously validated for analysis of cortisol in cat hair was used^35^. The minimum concentration of cortisol that could be distinguished from 0 was 0.007 µg/dL, and for samples with cortisol concentrations greater than 3.0 µg/dL, dilution was recommended. Results were converted from µg/dL to pg/mg hair^15^.

Hair from 13 cats and three 96-well plates were used to evaluate assay performance. Mean within-assay variation (CV) was determined on ten samples with low (2–3.6 pg/mg, three samples), medium (5.4–6.5 pg/mg, three samples) and high (8.7–85 pg/mg, four samples) cortisol concentrations, run in duplicates on one plate. Mean between-assay variation (CV) was determined by using three samples run in quadruplicates on three plates. For linearity and recovery upon dilution, one feline sample with high HCC was serially diluted with the assay diluent provided by the manufacturer 1:1, 1:1.33, 1:2, 1:4, 1:8, 1:16, and 1:32, and analyzed in triplicates on one plate. Recovery ([observed concentration/expected concentration]*100) was calculated. For recovery upon addition, three feline samples were spiked 1:1 in three different combinations (low and high sample, low and medium sample, low sample and high calibrator, as provided by the manufacturer) and analyzed in triplicates on one plate. To determine cortisol concentration, non-spiked samples from the three cats were also analyzed. Expected concentration ([concentration of non-spiked sample + concentration of the added sample or calibrator]*0.5) was calculated.

To evaluate stability of cortisol in cat hair over time, hair from two cats were prepared and the cortisol concentration was analyzed after 1, 8, and 24 months of storage in aluminum foil. For stability of extracted cortisol under deep frozen conditions, frozen reconstituted samples from the same two cats were re-run 8 and 24 months after initial cortisol analysis.

All individual samples were run in duplicates on a total of ten 96-well plates. In case of high CV (≥ 10%) between duplicates that could not be explained by low concentrations, samples were re-run. Samples with the lowest CV proceeded to data analysis. Samples exceeding the highest assay limit (3 µg/dL = 240 pg/mg) were set to 240 pg/mg.

#### Data analysis

In the case of missing or conflicting answers for any of the variables of interest, the respondent was contacted, and the answers were then adjusted, or the cat excluded accordingly. Cat age at the time of hair sampling was set to January 1st of each year. Cat sex was defined as spayed/neutered female or male, intact female or male, and females that, regardless of neutering status, had experienced pregnancy or estrus 2–16 weeks preceding hair sampling. For cats that were sampled in connection with neutering or spaying procedures, without experience of pregnancy or estrus, sex was set as intact. For owner-assessed stress, cats were assigned three different stress groups; no stress (no experience of potential stressors and no display of signs of stress), experience of potential stressors (but not displaying signs of stress), and displaying signs of stress. Cats that had experienced pregnancy or estrus were included in the group of cats displaying signs of stress, regardless of the owners’ assessment of stress signs. Cats in an owner-reported state of diabetic remission were included in the healthy group. For a summary of the questions and grouping of answers, see Table 1.

Data analysis was performed using R ^50^ and Minitab ^51^. Data was checked for normality using frequency distribution (histogram) and the Shapiro–Wilk normality test. Means and standard deviations were used when normality could be assumed, and median and inter-quartile ranges (IQR) were utilized when this was not the case. For evaluation of the relationship and differences of HCC between the three different hair sampling locations and methods, Spearman´s rank correlation and Bland–Altman plots were used, with 95% confidence intervals. Bland–Altman plots were interpreted graphically and by calculating the mean difference between paired values (bias). In cases where markedly differing datapoints, introducing a larger bias to the plot, were identified, they were removed prior to plotting. Limits of agreement (LOA, bias ± 1.96 SD) were constructed based on the standard variation (SD) of the differences between paired measurements. Multiple linear regression was used for evaluation of associations between HCC and owner reported cat characteristics, stress or health status. Two regression models were built; one with HCC quantified from clipped front leg samples as dependent variable, and one with HCC derived from combed samples as dependent variable. To improve model fit, the outcome variable HCC was log transformed for both regression models. Univariable linear regression was used for selection of variables, including variables with p < 0.2 in further model building. Based on previous studies^35,43^, the variables health status, presence of other animal species in the household, occurrence of potential stressors and signs of stress were included in building of the multivariable regression model, regardless of univariable regression outcome. The final regression model was decided with a backwards elimination process, where a lowered Akaike information criterion (AIC) was combined with exclusion of variables with p ≥ 0.05. Confidence intervals were set to 95% with a significance level of 5%. Quantile–quantile plots (QQ plots) and normality tests were used to assess regression residuals for normality. Biologically plausible interactions were included, and possible confounders were controlled in each regression model.

## Results

### Cats and general characteristics

**Fig. 1:**
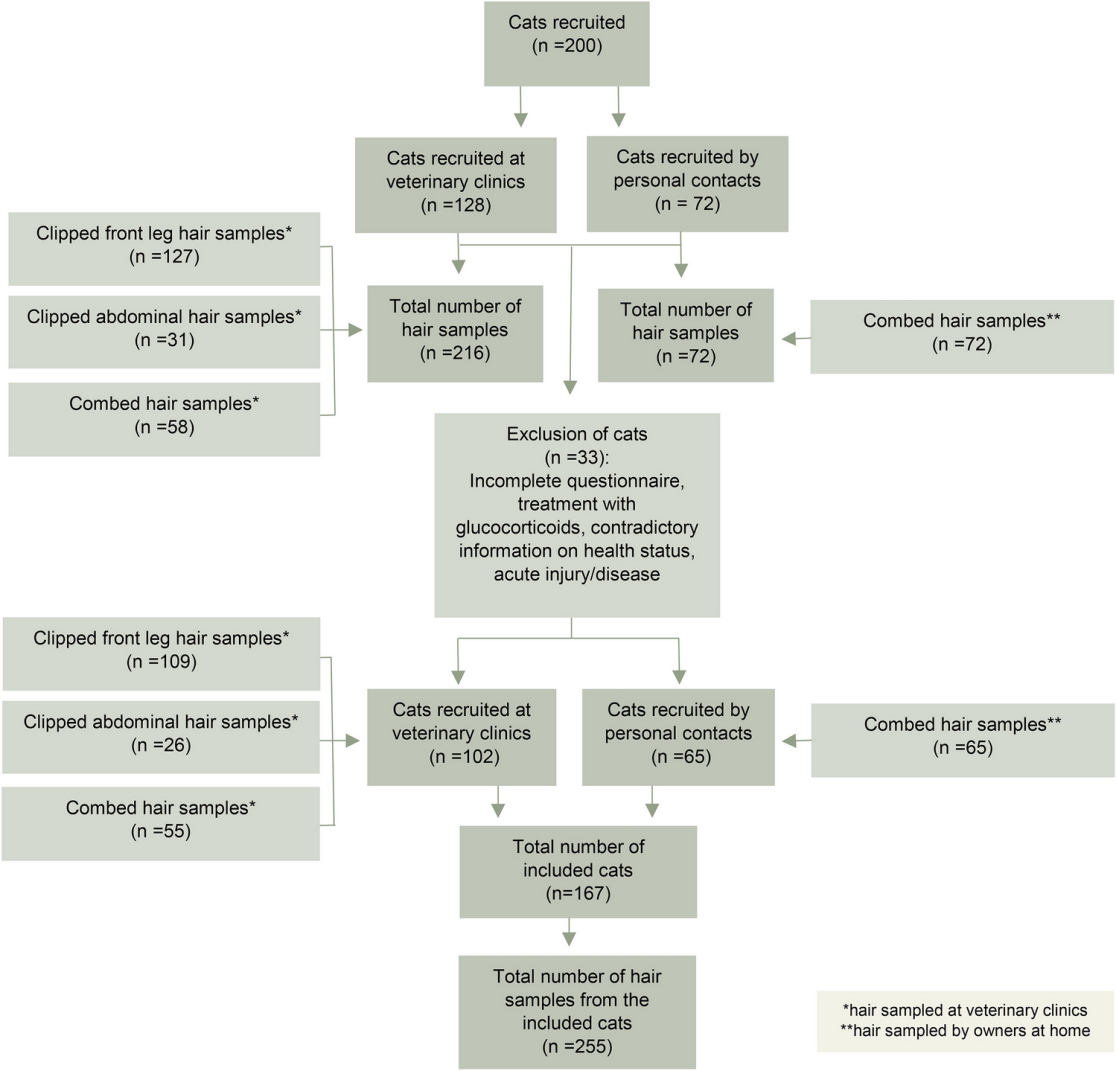
Overview of cats recruited and included in the study, including the different methods and body locations from where hair was sampled from the cats. Not all cats were sampled from all three locations.

In total 200 cats were recruited. Of these, 33 were excluded due to regular treatment with glucocorticoids, incomplete or obviously misinterpreted questionnaire answers, acute injury or disease, or contradictory information on health status. Complete questionnaires and hair from 167 cats remained for analysis (Fig. 1). One-hundred and three cats were reported as healthy, and 64 cats as chronically ill.

In healthy cats (n = 103), median age at sampling was 3 years (IQR 2–8). The majority of cats were spayed/neutered (63%), 15.5% were intact females, 5% were intact males, and 16.5% were females that, irrespective of neutering status at the time of sampling, had experienced pregnancy or a period of estrus.

In cats with chronic disease (n = 64), median age at hair sampling was 14 years (IQR 11–16). All cats were spayed or neutered, and no cat had experienced pregnancy or estrus. Type of chronic illness included one or more of the following conditions; diabetes mellitus (n = 28), hyperthyroidism (n = 19), chronic kidney disease (n = 14), hypertension (n = 5), arthritis (n = 3), chronic pancreatitis (n = 1), inflammatory bowel disease (n = 1), asthma (n = 1), chronic rhinitis (n = 1), allergy (n = 1), and tooth resorption (n = 1).

### Assay performance

Mean within assay CV was 26.7% for low, 11.5% for medium and 10.1% for high concentrations, respectively. Mean within-assay CV for all analyzed 10 samples was 15.5%. Mean between-assay CV was 7.6% for concentrations 5.9–30.1 pg/mg. To determine recovery after dilution, a pooled sample with known concentration of cortisol (25.9 pg/mg) was diluted 1:1/3, 1:2, 1:4, 1:8, 1:16 and 1:32. Recovery after dilution was adequately linear, and was 109%–460%, where the most diluted sample represented the highest recovery percentage. Recovery upon addition was 80%-85% for concentrations 8.5–134 pg/mg.

A higher consistency was seen between original and re-run frozen reconstituted samples, compared to original samples and samples prepared from hair that had been stored in aluminum foil, see Table 2.

**Table 2:** Stability of cortisol concentrations in two cats (A and B), in samples where cortisol was extracted from hair stored at room temperature (RT), and in samples where cortisol was reconstituted in a phosphate buffer (PBS) and then stored at –80°, for 0–1, 8 and 24 months respectively before cortisol quantification.

	**Storage method**	**0–1 months of storage**	**8 months of storage**	**24 months of storage**	**Mean**	**SD**
Cat AHCC (pg/mg)	Hair in RT*	11.2	8.0	12.8	10.7	2.4
	Extraction in PBS** at – 80°	11.2	9.6	12.8	11.2	1.6
Cat B HCC (pg/mg)	Hair in RT*	18.0	12.0	12.3	14.1	3.4
	Extraction in PBS** at – 80°	18.0	17.3	18.8	18.0	0.75

*Hair stored in aluminum foil at room temperature (RT) until extraction of cortisol and cortisol quantification, **Extracted cortisol in a phosphate buffer (PBS), all samples prepared and frozen at the same time, and stored at –80° until cortisol quantification

### Hair samples and hair sampling location and sampling method

A total of 255 hair samples were collected (see Fig. 1). Median HCC in clipped samples from the front leg was 5.6 pg/mg (IQR 4–7.2, n = 109), in clipped samples from the abdomen 4.8 pg/mg (IQR 3.4–8, n = 26) and in samples obtained through combing 7.2 pg/mg (IQR 4.8–11.5, n = 120). The HCC range was widest in samples obtained from combing (2.4–240 pg/mg) compared to front leg (1.4–134 pg/mg) and abdomen (1.6–13.6 pg/mg).

**Fig. 2:**
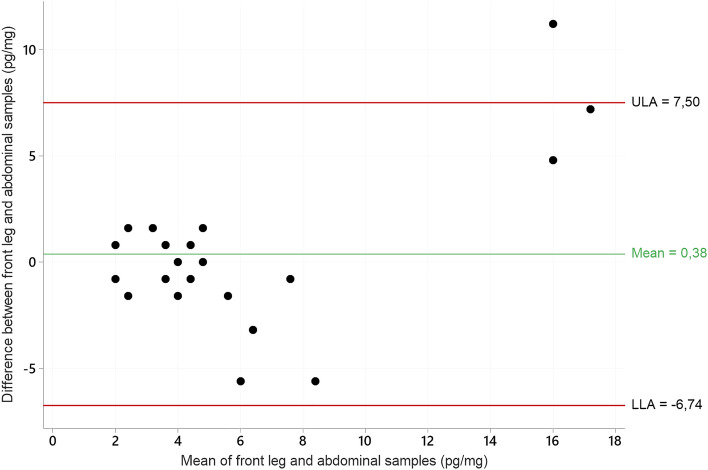
Bland–Altman plot of agreement between hair cortisol concentrations (pg/mg) in hair samples from the dorsal area of the front leg and the ventral area of the abdomen in cats (n = 23). ULA = upper limit of agreement, LLA = lower limit of agreement.

Twenty-three cats had hair sampled from both the front leg and from the abdomen, with a Spearman´s Rho of 0.72 (CI 0.39–0.89, n = 23). In Bland–Altman analysis, the mean difference between paired HCC was 0.38 pg/mg and the limits of agreement was -6.74 and 7.50 (LOA, 0.38 ± 1.96 SD). One observation was beyond LOA. Agreement between measurements was higher for lower HCC concentrations (mean ≤ 5.6 pg/mg) than for higher concentrations, see Fig. 2.

**Fig. 3:**
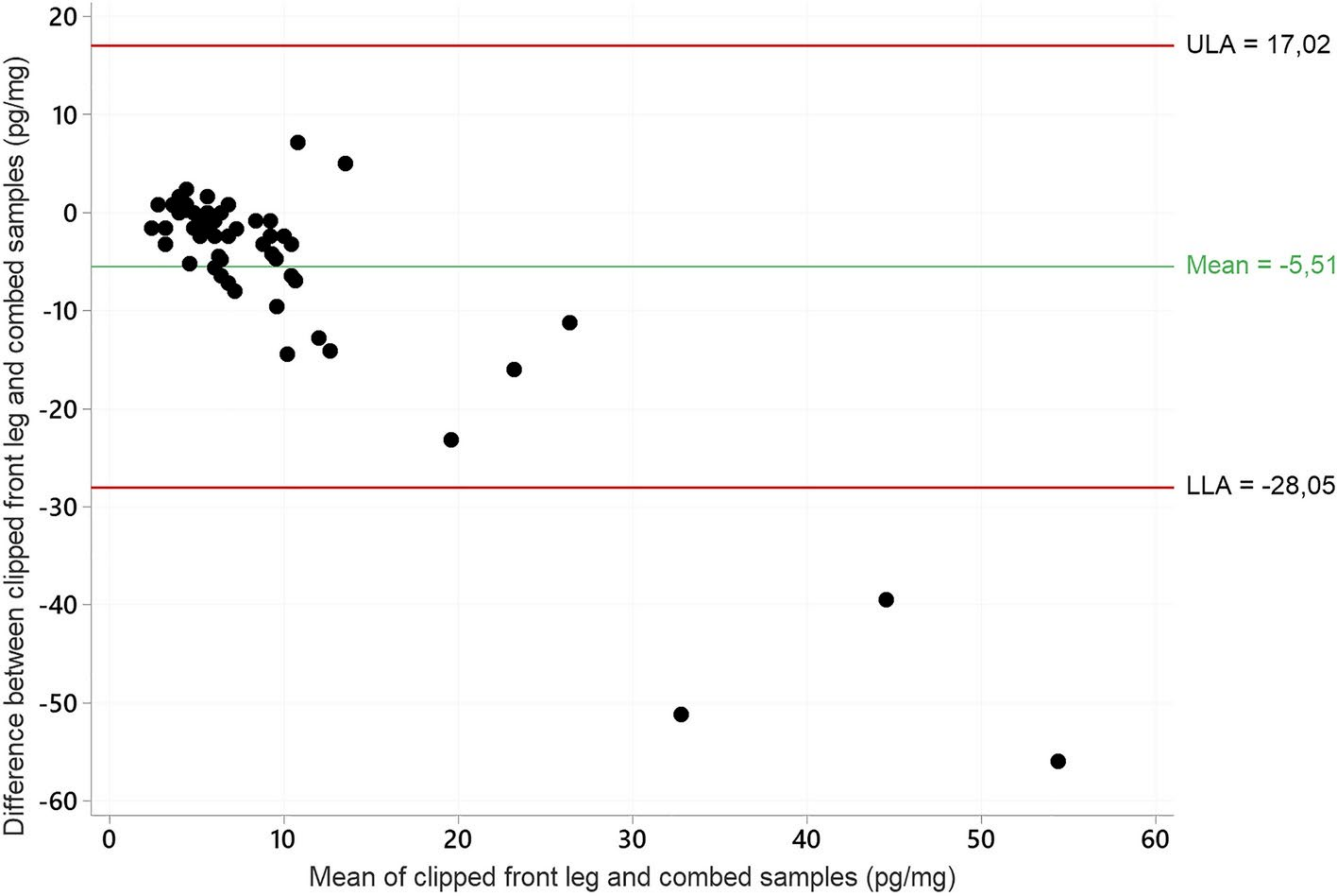
Bland–Altman plot of agreement between hair cortisol concentrations (pg/mg) in hair samples from the dorsal area of the front leg and combed samples in cats (n = 59). ULA = upper limit of agreement, LLA = lower limit of agreement.

Sixty-two cats had hair sampled from both the front leg and by combing, with a Spearman´s Rho of 0.61 (CI 0.41–0.76, n = 62). For the Bland Altman plot, three observational outliers were removed – see Fig. 3. The mean difference in paired values was -5.51 pg/mg and the limits of agreement was -28.05 and 17.02 (LOA, -5.51 ± 1.96 SD, n = 59). Three observations were beyond LOA. As for the comparison between clipped front leg and abdominal samples, agreement between measurements was highest in lower HCC concentrations (mean ≤ 10 pg/mg) and decreased with increasing HCC.

### HCC and associations with cat characteristics, health status and stress

Regression analysis with HCC quantified from clipped front leg samples comprised every included cat with front leg samples (n = 109). From univariable linear regression models, sex (p = 0.18) and multi-cat household (p = 0.04), were included in building the multiple linear regression model. Further, the variables health (p = 0.228), presence of other animal species in the household (p = 0.750), occurrence of potential stressors (p = 0.503), and presence of signs of stress (p = 0.270), were included ^35,43^. In the final linear multivariable regression model, the only factor remaining significant for prediction of HCC were sex and multi-cat household. Females that had experienced pregnancy or heat had higher HCC than spayed females (p = 0.008), and cats that lived in a household with at least one more cat had higher HCC than cats in single cat household (p = 0.042). See Table 3. and 4 for details.

**Table 3.** Overview of variables included in multivariable linear regression analysis of hair cortisol concentrations (pg/mg) from hair samples collected by clipping the front leg (n=109) or by combing the dorsum and lateral sides (n=120) of the cat (n=167). Note that 62 cats were sampled in both ways and are thus represented in both groups. Hair cortisol concentrations were log-transformed for regression analysis.

Variable		Clipped front leg hair samples (n=109)	Combed hair samples (n=120)
Sex and neutering status*	Neutered male	34	58
	Spayed female	41	34
	Intact male	5	2
	Intact female	13	11
	Female with experience of estrus/pregnancy	16	15
Number of cats in household*	Single-cat household	47	61
	Multi-cat household	62	59
Presence of other animals in household (e.g., dog)**	No	95	101
	Yes	14	19
Presence of chronic disease* **	No	67	83
	Yes	42	37
Exposure to potential stressors**	No	49	58
	Yes	60	62
Display of signs of stress**	No	85	94
	Yes	24	26

*Variables were selected for multiple regression analysis if p < 0.200 in univariable analysis

**Variables were selected for multiple regression analysis regardless of univariable outcome, based on results from previous studies

**Table 4:** Back-transformed predictions of hair cortisol concentrations (HCC) in two final linear regression models, where hair was sampled by clipping the dorsal area of the front leg (n = 109), and by combing the dorsum and lateral area of the thorax and abdomen (n = 120) in the cat.

	**HCC measured in hair clipped from the dorsal area of the front leg**			**HCC measured in hair combed from the dorsal and lateral areas of the body**		
	**Change in HCC (%)**	**CI (%)**	**p**	**Change in HCC (%)**	**CI (%)**	**p**
**Outcome and variables**						
*Sex*						
*Intact female vs spayed female*	56	-7 – 158	0.085	89	-4 – 274	0.066
*Intact male vs spayed female*	125	-6 – 439	0.068	499	46 – 2356	0.013 *
*Female with experience of pregnancy or heat vs spayed female*	76	16 – 167	0.008**	84	-0.2 – 239	0.051
*Chronic disease vs healthy*	49	-0.9 – 124	0.055	61	6 – 144	0.025*
*Multicat household*	38	1 – 89	0.042*	-	-	-
*CI; confidence interval*						

Regression analysis with HCC quantified from combed hair samples comprised every included cat with combed samples (n = 120). From univariable linear regression models, sex (p = 0.102) and health (p = 0.190), were included in building the multiple linear regression model. As previously mentioned, the variables presence of other animal species in the household (p = 0.737), occurrence of potential stressors (p = 0.311), and presence of signs of stress (p = 0.989), were included^35,43^. In the final linear multivariable regression model, factors remaining significant for prediction of HCC were sex and health. Intact males had higher HCC than spayed females (p = 0.013) and cats with chronic disease had higher HCC than healthy cats (p = 0.025). See Table 3. and 4 for details.

## Discussion

This study measured cortisol concentrations in hair from domestic cats, sampled by two different methods and from three different body locations. HCC varied with and within different body locations and sampling methods. Despite this, agreement between methods was found, and both sex and health were associated with HCC. Being a female with experience of pregnancy or estrus or living in a multi-cat household was associated with elevated HCC in hair sampled from the front leg. Presence of chronic disease was associated with increased HCC in combed hair samples, as was being an intact male. Exposure to potential stressors or displaying signs of stress were not associated with changes in HCC. Based on the results of the present study, storing samples as reconstituted samples is preferable for HCC quantification compared to storing hair.

The common practice in hair cortisol analysis involves initially shaving a specific area and then repeating the process after a designated period of hair regrowth, known as the shave-reshave method. This ensures the inclusion of actively growing hairs in the sample, aligning with the time frame of interest, as cortisol is incorporated into actively growing hair strands^23,52^. Differences in cortisol concentrations among hair from different body regions have been seen in various species^22,24,35,53^. Therefore, it is recommended to sample hair from one single body region when measuring HCC. In the present study, sampling areas were chosen based on convenience in a clinical setting and to minimize sampling-associated stress for the cat. The aim was to retrospectively study a time frame of 2–16 weeks, with a questionnaire designed to cover the cat’s history during this period in relation to hair sampling.

The specific properties of cat hair have to be considered when using HCC. Hair growth varies with different seasons and photoperiods^34,49^, factors that were not investigated in the present study. A slower growth rate could result in prolonged cortisol incorporation in the hair, leading to higher concentrations of cortisol, and representing a longer retrospective time for cortisol incorporation. The cat pelage includes primary (guard) and secondary (wool) hairs, and their distribution over the body, their growth rate and follicular activity varies^34^. These factors, as well as shedding patterns^34^, and varying hair lengths across different body parts, could have contributed to the variations in HCC seen between different body locations in the present study.

Given the differences in both body location and retrieval method, different HCC between the clipped and combed hair samples was expected. This expectation was confirmed, with the highest variation observed among the combed samples. The influence of a few individuals with very high HCC, predominantly measured from combed samples, could explain some of this variation. Additionally, the broader area of the body from which hair strands are sampled during combing, as opposed to the front leg, may have introduced further variability. Combing involves sampling a large proportion of loose hairs, which could make this method more susceptible to seasonal and individual shedding, and to grooming behaviors. No information on grooming behavior was collected in the present study. Combing may include more skin material in the hair sample, but thorough washing prior to cortisol quantification minimizes external contribution to HCC variability^23^, and skin material is thus not likely contributing to the wide HCC range with this method. For clipped front leg samples and combed samples, there was consistency in paired measurements, especially for lower HCC. The same pattern of consistency was also evident in paired measurements from hair clipped from the front leg and the abdomen. Similar associations between HCC and cat characteristics and health were found between clipped front leg samples and combed samples, though not all statistically significant at p < 0.050. It is important to note that not all cats were sampled using both methods and from all three locations, and a larger sample size would have been beneficial for comparison. This highlights the challenges faced when collecting samples for research in a clinical setting. Combing is a less standardized method of hair sampling. However, avoiding repeated sampling is beneficial for increasing compliance among cat owners, and hair sampled by combing has potential as an owner- and cat-friendly method of hair sampling for HCC analysis.

Presence of chronic disease was associated with higher HCC, compared to healthy cats. Previously, chronically elevated cortisol levels, as measured in HCC, have been associated with dermatophyte infection in shelter cats^43^. A higher urine cortisol-creatinine ratio has been described in cats with hyperthyroidism^54^ and in hospitalized cats with various diseases^55^, as well as in shelter cats with signs of systemic disease ^12^. Higher cortisol concentrations could be associated with both the disease process^54^, and with events that are related to, but not directly associated with disease. This may include veterinary visits and transportation of the cat to the clinic^56^, cat synchronization with owner stress^57^, and daily administration of medications. Although hyperthyroidism, chronic kidney disease, and diabetes mellitus were the most commonly represented diseases in the studied population, a diverse range of diseases was observed, and the cats exhibited various stages of illness. Further examination of specific diseases could provide deeper insights into the influence of individual diseases and disease states on HCC. Interestingly, the presence of clinical signs reported by owners did not show an association with HCC. This discrepancy could be attributed to factors such as questionnaire design, inaccurate owner reporting, or the owners’ ability to recognize clinical signs of disease. Furthermore, HPA-axis dysregulation, manifesting as hypocortisolism following a period of chronic HPA-axis hyperactivity, might contribute to this observed discrepancy^58^. The possibility of a disparity between the observable condition of a chronically ill cat and its actual impact, as measured by HCC, warrants additional investigation.

Our results reveal a sex-associated difference in HCC. In clipped front leg hair, females with reported experiences of pregnancy or estrus, had higher HCC than spayed females. In combed hair, intact males had higher HCC than spayed females. Suggested causes for effect of sex on HCC in different species include the influence of social rank^59^, territorial behavior^60^, differences in body condition^25^, and effect of gonadal steroids on HPA-axis activity ^61^. During pregnancy, cortisol is crucial for fetal development, leading to increased circulating cortisol concentrations until parturition^31,62^. A lower HCC has been reported in spayed free-roaming domestic female cats compared to intact females, possibly influenced by agonistic behavior or reproductive status^37^. The associations in our study may result from hormonal changes during pregnancy, parturition, increased energetic expenditures, and general maternal efforts post-partum^38,63^. The majority of intact females (15/16) without owner-reported estrus or pregnancy were at least one year old at the time of hair sampling, suggesting potential estrus experiences not reported by the owner ^64^. In a study of wildcats and feral cats, higher HCC was observed in male cats compared to females, although this difference was not statistically significant, possibly due to a small sample size^65^. In our study, we found an association between male sex and HCC. However, similar to the previous study, the limited number of cats in our sample influenced the results, with uncertainty highlighted by a wide confidence interval. A larger sample size is necessary for a more comprehensive exploration of HCC and sex in domestic cats.

In the present study, no association between HCC and exposure to potential stressors or display of stress-related behaviors were seen. Previously, HCC in cats has been positively associated to defecating or urinating outside the litterbox^35,36^ and to displaying aggressive behavior towards family members ^36^, and negatively associated to a groomed and soft hair coat^35^. In one of these studies, owner-reported stress levels were not significantly associated with HCC when other factors related to the cat and its home environment were considered ^35^. An explanation for the lack of association between stress and HCC may be failure of owners to recognize signs of stress in their cats ^11^, or variability in HPA-axis activity related to individual stressor response ^58,66,67^. In the present study, no consideration of different cat temperaments and personalities were made, which could affect both stress coping mechanisms and stress responses ^3,68^.

Cats living in multi-cat households had higher HCC than cats in single cat households. A multi-cat household was defined as the cat sharing living space with at least one other cat, and in the data analysis no differentiation on the total number of cohabitating cats were made. In a multi-cat household, cats need to share resources, with potential emergence of inter-cat aggression and other stress-linked behaviors ^69^. However, a multitude of potential factors influence cat behavior in both single and multi-cat households ^7,46^.

This study has several limitations. First, this is an observational study, and as such, there is limited control over the included subjects. The cat population at large is probably well represented, but confounding factors may go undetected. The sample size is small, and the hair sampling performed at home by owners lacked standardization, introducing variability into the collection process. Also, reliance on owner obtained information about the cats may introduce subjectivity and potential inaccuracies, including recall bias and misinterpretations. No clinical confirmation of the cats’ health status or other related information was available. The lack of a validated stress scale or evaluation tool could have limited the accuracy of stress assessment.

Chronic stress has profound implications for feline welfare. Given that stress often goes unnoticed by owners, there is a need for methods to facilitate stress evaluation in cats. The quantification of HCC holds promise for a non-invasive aid in assessment of long-term HPA-axis activity in felines. This study explored a novel, low-stress hair sampling method for cortisol quantification. Although much remains unknown about the factors influencing HCC, samples obtained by combing demonstrate potential for simplified sample collection and assessment of HCC in relation to various cat characteristics. Despite variations between the studied sampling methods, a concordance between them was observed, and associations between HCC and sex and health was seen. Combing showed potential as a cat-friendly sampling method, addressing the challenges encountered in sample collection within a clinical setting. The limitations of this study warrants caution in interpreting results. However, the findings contribute to the potential use of a low-stress combing method for HCC assessment, emphasizing the need for research to further explore this approach.

## Data Availability

The datasets generated during and/or analyzed during the current study are available from the corresponding author on reasonable request.
